# Effect of crude leaf extract of *Osyris quadripartita* on *Plasmodium berghei* in Swiss albino mice

**DOI:** 10.1186/s12906-015-0715-3

**Published:** 2015-06-16

**Authors:** Senait Girma, Mirutse Giday, Berhanu Erko, Hassen Mamo

**Affiliations:** Department of Microbial, Cellular and Molecular Biology, College of Natural Sciences, Addis Ababa University, P.O. Box 1176, Addis Ababa, Ethiopia; Aklilu Lemma Institute of Pathobiology, Addis Ababa University, P.O. Box 1176, Addis Ababa, Ethiopia

**Keywords:** Antimalarial activity, *Osyris quadripartita*, *in vivo*, *Plasmodium berghei*, Swiss albino mice, Ethiopia

## Abstract

**Background:**

Continuous emergence of multi-drug-resistant malaria parasites and their rapid spread across the globe warrant urgent search for new anti-malarial chemotherapeutics. Traditional medicinal plants have been the main sources for screening active phytochemicals against malaria. Accordingly, this study was aimed at evaluating the anti-malarial activity of *Osyris quadripartita* Salzm. Ex Decne., a plant which is used for traditional malaria treatment by local people in different parts of Ethiopia.

**Methods:**

Aqueous, chloroform and methanol crude leaf extracts of the plant have been prepared and tested for acute toxicity and anti-malarial efficacy in *Plasmodium berghei* (ANKA strain)-infected Swiss albino mice.

**Results:**

At three oral doses of 200, 400 and 600 mg/kg the plant material was safe, chemosuppressive and thus prevented body weight loss, hematological abnormalities and increased mice mean survival time compared to the negative control. The most efficacious extract was that of chloroform which prolonged mean mouse survival past day 11 of infection with all the mice in this group having the highest parasitemia suppression rate (41.3 %, at 600 mg/kg) although parasite clearance was not achieved compared to the standard drug (chloroquine) against the parasite.

**Conclusion:**

The finding supports the traditional use of the plant for the treatment of malaria. However, further confirmatory studies followed by isolation and characterization of the active anti-malarial compound (s) of the plant that is/are responsible for the observed parasite suppression is needed before it is recommended for malaria drug search and discovery.

## Background

Though malaria mortality rates have fallen by 47 % globally and by 54 % in the World Health Organization (WHO) African Region since 2000, about 198 million cases and 584 000 deaths occurred in 2013 showing that malaria is still among the leading human infectious diseases with 90 % of the cases and deaths occurring in Africa [1]. In Ethiopia, although evidences suggest a substantial decline in malaria cases and deaths in recent years compared to the baseline year of 2004, the occurrence of several isolated outbreaks was reported nationwide and the disease remains a major cause of outpatient consultation in the country [2].

Due to climatic changes and coupled-human-natural factors malaria epidemiology is progressively changing putting more people at-risk. Moreover, there is a rapid emergence of drug-resistant *Plasmodium* strains. For instance, resistance has already been developed against the latest first-line anti-malaria drug, artemisinin, in Asia [3]. Malaria control efforts are further complicated by the increased resistance of mosquito vectors to insecticides [4] together with challenges of having effective anti-malaria vaccines. Thus, there is urgent need to search for effective, easily available, affordable and safe alternative anti-malaria drugs that can be integrated into the existing malaria control interventions to successfully curtail the disease and for its eventual elimination or eradication.

It is well-known that plants have been and are still the mainstay of traditional medicine against malaria and other diseases in resource-limited settings as over one-third of the population in such countries lack access to essential medicines [5]. However, the claimed potency of medicinal plants has to be scientifically evaluated and toxicological studies should be done. Rigorous *in vitro* and *in vivo* toxicological investigations are required to determine the type and degree of toxicity, safety and efficacy of plant products in malaria drug research and ultimate discovery as well as to recommend or discourage a plants’ traditional medicinal use.

To this end, various studies have been conducted to evaluate the safety and anti-malarial efficacy of traditionally used plants in Asia and Africa. For example, Ramazani *et al.* [6] worked on ten Iranian plant species but only - *Boerhavia elegans*, *Solanum surattense* and *Prosopis juliflora –* showed a promising anti-plasmodial activity *in vitro* and *in vivo* with no toxicity. Verma *et al.* [7] reported that *Holarrhena antidysenterica* and *Viola canescens* exhibited *in vitro* anti-plasmodial activity in Himalaya. From sub-Saharan Africa in Burkina Faso [8], promising *in vitro* anti-plasmodial results were obtained for the plant *Dicoma tomentosa* with the dichloromethane, diethylether, ethylacetate and methanol extracts demonstrating a high activity. In the same study hot water and hydroethanolic extracts also showed a good activity, which was also confirmed *in vivo* for all tested extracts.

Despite their wide use in the traditional healthcare, the work that has been done to evaluate the safety and efficacy of Ethiopian traditional medicinal plants is relatively less extensive. A number of traditional medicinal plants remain unevaluated. *Osyris quadripartita* which is commonly used in Ethiopian traditional medicinal practices [9–12] and elsewhere in Africa [13] is a case in point. Specifically, the leaf of this plant is used to traditionally treat malaria [14, 15] in Ethiopia and cancer in Algeria [16]. The anti-inflammatory property of a methanol extract of *O. quadripartita* was demonstrated in effectively reducing increased capillary permeability induced in rats by various chemical mediators [17]. The antioxidant effect of this plant species has also been evaluated and was found to be good [18]. The plant has anti-bacterial and anti-fungal activities [19]. According to this same work, the plant was active against Gram-negative bacteria such as *Escherichia coli* and *Pseudomonas aeruginosa* as well as Gram-positives like *Staphylococcus aureus*. Though its effect was relatively lesser compared to other parallel test extracts the plant crude extract had a comparable efficacy to that of the positive control (gentamicin sulphate). The anti-bacterial and anti-fungal effects of *O. quadripartita* were confirmed by a latter more comprehensive study [20]. A different species within the genus, *Osyris alba,* which was tested against *Entameba histolytica*, *Giardia intestinalis* and *Candida albicans* has shown a reasonable effect [21].

Phytochemical studies on the genus *Osyris* has yielded hexyl and hexenyl derivatives, sesquiterpenes, phenolic acids, flavoinoids, pyrrolizidine and quinolizidine alkaloids, long chain hydrocarbons and fattyacids, triterpenes, dihydro-β-agarofuran sesquiterpenes, phenolics phenyl propanoids and lignans [17, 21–26]. However, the possible anti-plasmodial effect of the genus was not tested *in vivo* and *in vitro* either. The aim of this study was, therefore, to assess the anti-plasmodial activity and safety threshold of *O. quadripartita* in Swiss albino mice in an attempt to contribute towards screening traditional medicinal plants for malaria control.

## Methods

### Plant description

The genus *Osyris* which includes more than 34 species belongs to the family Santalaceae and the species *O. quadripartita* Salzm. ex Decne*.* (locally called *qeret* in Amharic and *wato* in Afaan Oromoo) is an evergreen, dioecious tree or shrub reaching a height of 1–7 m with many branches and the branches sometimes pendant [27]. It is hemiparasitic and sometimes opportunistically taps into the root systems of nearby plants and parasitizes them although it can freely grow and survive [28, 29]. The plant which is native to Africa, southwestern Europe and Asia is commonly known as wild tea plant [30]. This plant is widely distributed in Ethiopia [27].

### Sample collection

Leaf samples of *O. quadripartita* were collected in December 2013 from its natural habitat in *Wertu kebele* around Fiche Town, some 110 km north of Addis Ababa. Previous ethnobatanical studies characterized Fiche and its flora in relation to traditional medicinal values [31, 32]. The collected plant sample for this study was identified and authenticated by the second author who is an ethnobotanist at Aklilu Lemma Institute of Pathobiology, Addis Ababa University (AAU) and the voucher specimen was deposited in the National Herbarium of AAU with voucher number designated as Senait 001/2014.

### Preparation of crude plant extracts

The leaf tissues were washed thoroughly with running tap water and each plant material was reduced to smaller fragments. The washed leaves were air dried at a room temperature under shade in the Biomedical Sciences Laboratory, Department of Microbial, Cellular and Molecular Biology (DMCMB); College of Natural Sciences (CNS), AAU and were ground into fine powder using an electric grinder and kept in a tightly closed brown bottle until used for extraction.

The leaf crude extracts of aqueous, 98 % methanol and chloroform were prepared by the established cold maceration technique [33]. Briefly, a 100 g plant extract material was refluxed in 1000 ml of aqueous, methanol or chloroform and the respective mixtures were placed on an orbital shaker (GFL, model 3020, Germany) at 160 revolutions per minute (rpm) for 72 hours. The mixture was first filtered using cotton and then the filtrate was passed through Whatman filter paper №3, 15 cm pore size with retention down to 0.1 μm in liquids (Whatman LTD, England). The methanol and chloroform extracts were concentrated in a rotary evaporator (Buchi type TRE121, Switzerland) at a temperature of 45 °C; whereas the aqueous extract was freeze-dried using centrifugal freeze dryer (Model 5 PS, Christ, England). All the extracts were stored in screw cap vials at −20 °C until used for the *in vivo* experiment.

### Acute toxicity test

The aqueous and methanol extracts of the plant material were dissolved in 10 ml of distilled water (dH_2_O) and that of chloroform was dissolved in 3 % Tween (T)-80 prior to the actual experiment, the chloroform extract failed to dissolve in dH_2_O. Individual body weights (BW) of mice were determined and recorded shortly before and after the plant was administered to evaluate its toxicity as per the Organization for Economic Co-operation and Development (OECD) *Guidelines for the Testing of Chemicals* [34]. For this purpose non-infected female mice aged 6–8 weeks (mean age 7.4 weeks) and weighing 23-38 g (mean weight 28.35 g) were used.

Twenty mice were used by randomly dividing them into four groups of 5 mice per cage. The mice were starved for 3–4 hrs before the experiment began with only water allowed and 1–2 hrs after the extracts were given. Then, the mice in groups 1, 2 and 3 were orally given 1000, 1500 and 2000 mg/kg BW in single dose volume of 0.2 ml of the extract, respectively. The mice in the negative control group received 0.2 ml of dH_2_O or T-80. Then, the mice were monitored continuously for 1 hr, intermittently for 4 hrs and for a period of 24 hrs for any gross behavioral changes such as rigidity, sleep, mortality and other signs of acute toxicity manifestations, and the follow-up continued for 28 days [35].

### Parasite inoculation and anti-malarial assays

#### Parasite inoculation and maintenance

When working on chloroquine (CQ)-sensitive *P. berghei* CQ is employed as a standard positive control [36] and in this study a similar study design was followed. Previously maintained Swiss albino mice 5–7 weeks of age were used for the test. CQ-sensitive strain of *P. berghei* (ANKA strain) previously maintained in the DMCMB animal house were used for the experiment. Female mice having variable parasitaemia level were used as donor animals. The parasitaemia of the donor mice was first determined and parasitized erythrocytes were obtained by cardiac puncture using ethyl ether as anesthesia and sodium citrate (0.5 %) diluted in physiological saline (0.9 %) as an anticoagulant. The dilution was made based on the parasitaemia of the donor mice and the erythrocyte count of normal mice in such a way that 1 ml blood contains 5x10^7^ infected erythrocytes [37]. Each mouse was inoculated by intra-peritoneal injection with a blood suspension (0.2 ml) containing 1x10^7^ parasitized erythrocytes. The parasite was maintained by serial passage of blood from infected mice to non-infected ones on a weekly basis.

#### Anti-malarial activity

The standard four-day suppressive method was used in screening of the plant extracts [36]. The experimental mice infected with 1x10^7^*P. berghei* each were randomly divided into nine test groups and three control groups (each for CQ as the standard anti-malarial drug and dH_2_O or 3 % T-80 as negative controls (NC). The test extracts were prepared in three different doses (200, 400 and 600 mg/kg BW and CQ at 25 mg/kg, and all including the vehicles were given in a volume of 0.2 ml per mouse. The extracts were administered as a single dose per day. Both the extract and the drug were given through intra-gastric route by using a standard intra-gastric tube to ensure safe ingestion.

#### Chemosuppression

Treatment was started after 3 hrs of infection with *P. berghei* on day 0 (D0, inoculation day) and was continued daily for four days (*i.e.* from D0 to D3). On the fifth day (D4) a blood sample was collected from the tail snip of each mouse. Thin smears were prepared, stained with 10 % Giemsa solution and scanned under the light microscope with an oil immersion objective of 100x magnification power following established procedure [38]. Then, the percent suppression of each extract with respect to the control groups and the parasitaemia was determined by counting a minimum of five fields per slide. Percent of suppression (% suppression) and percent parasitaemia (% parasitaemia) were calculated in comparison to the control using the method described by the modified Peters and Robinson formula [39]:

$$ \% Suppression=\frac{\mathrm{parasitasmia}\kern0.5em \mathrm{in}\kern0.5em \mathrm{negative}\kern0.5em \mathrm{control}\hbox{-} \mathrm{parasitasmia}\kern0.5em \mathrm{in}\kern0.5em \mathrm{treated}\kern0.5em \mathrm{group}}{\mathrm{parasitasmia}\kern0.5em \mathrm{in}\kern0.5em \mathrm{negative}\kern0.5em \mathrm{control}}\mathrm{X}\kern0.5em 100. $$

$$ \%\kern0.5em  Suppression\kern0.5em =\frac{\mathrm{number}\kern0.5em \mathrm{of}\kern0.5em \mathrm{parasitized}\kern0.5em \mathrm{R}\mathrm{B}\mathrm{C}}{\mathrm{total}\kern0.5em \mathrm{R}\mathrm{B}\mathrm{C}\kern0.5em \mathrm{counted}}\mathrm{X}\kern0.5em 100. $$

#### Determination of BW change

The BW of each mouse in all the groups was measured before infection (D0) and on D4 in case of treatment, by using a sensitive digital weighing balance (A&D Company LTD, Japan) and mean BW per group was calculated using the formula:

$$ Mean\kern0.5em BW=\frac{\mathrm{mean}\kern0.5em \mathrm{body}\kern0.5em \mathrm{weightof}\kern0.5em \mathrm{mice}\kern0.5em \mathrm{in}\kern0.5em \mathrm{a}\kern0.5em \mathrm{group}\kern0.5em }{\mathrm{total}\kern0.5em \mathrm{number}\kern0.5em \mathrm{of}\kern0.5em \mathrm{mice}\kern0.5em \mathrm{in}\kern0.5em \mathrm{that}\kern0.5em \mathrm{group}}. $$

#### Determination of packed cell volume

Packed cell volume (PCV) was determined using blood collected from tail of each mouse in heparinized microhaematocrit capillary tubes (Globe Scientific Inc, Paramus, NJ, USA) and filled up to 3/4th of the tube with blood, sealed one end with crystal seal and placed with the open end of the tube to the center and the sealed end outwards of a microhaematocrit reader (Hawsksley & Sons LTD, England). The blood was centrifuged at 12,000 rpm for 5 min and then the volume of the total blood and the volume of erythrocytes were measured using a ruler. Measurement was done before infection and on D4 after infection. PCV was calculated using the formula described by Gilmour and Sykes [40]:

$$ \mathrm{P}\mathrm{C}\mathrm{V}=\frac{\mathrm{volume}\kern0.5em \mathrm{of}\kern0.5em \mathrm{total}\kern0.5em \mathrm{erythrcytes}\kern0.5em \mathrm{in}\kern0.5em \mathrm{a}\kern0.5em \mathrm{given}\kern0.5em \mathrm{volume}\kern0.5em \mathrm{of}\kern0.5em \mathrm{blood}}{\mathrm{total}\kern0.5em \mathrm{blood}\kern0.5em \mathrm{volume}}\mathrm{x}\kern0.5em 100. $$

#### Determination of mean survival time

Mortality was monitored daily and the number of days from parasite inoculation up to death was recorded for each mouse in the treatment and control groups throughout the follow-up period. The mean survival time (MST) for each group was calculated using the formula below:

$$ MST=\frac{\mathrm{sum}\kern0.5em \mathrm{of}\kern0.5em \mathrm{survival}\kern0.5em \mathrm{time}\kern0.5em \left(\mathrm{days}\right)\kern0.5em \mathrm{of}\kern0.5em \mathrm{mice}\kern0.5em \mathrm{in}\kern0.5em \mathrm{a}\kern0.5em \mathrm{group}}{\mathrm{total}\kern0.5em \mathrm{number}\kern0.5em \mathrm{of}\kern0.5em \mathrm{mice}\kern0.5em \mathrm{in}\kern0.5em \mathrm{that}\kern0.5em \mathrm{group}}. $$

#### Ethical considerations

The study was conducted after ethical clearance was obtained from CNS Institutional Ethics Review Board, AAU. The living area for mice allowed them to satisfy their basic needs including the ability to eat, drink, urinate, defecate and also regulate their body temperature. Overall there was a humane handling of the experimental mice throughout the study.

#### Data analysis

The PCV and BW of *P. berghei*-infected mice in each group that were treated with the same extracts and dose were compared between D0 and D4, the results were expressed as mean plus or minus the standard error of the mean (M ± SEM) by one way analysis of variance (ANOVA) and 2-tailed Student’s *t*-test using statistical package for the social sciences (SPSS) version 15 software (SPSS IBM, Chicago, IL, USA). The suppressive test results were analyzed by ANOVA followed by Tukey-multiple comparison test to compare the level of parasitaemia, and survival times of the infected mice between the control group and the extract treated groups at a fixed time. All the data were analyzed at a 95 % confidence interval. *P-value* less than 0.05 was considered as statistically significant and <0.01 as highly significant.

## Results

### Extraction yield

The methanol extract of the plant material was the highest yield followed by the aqueous and chloroform extracts (Table 1)*.* The differences between the yields were significant.

**Table 1:** Yield of aqueous, chloroform and methanol crude leaf extracts of*O. quadripartita*

Solvent	Plant powder (g)	Extraction solvent (ml)	Yield (g)	Yield (%)
Aqueous	100	1000	6.60	6.60*
Methanol	100	1000	32.75	32.75*
Chloroform	100	1000	1.447	1.447*

Key: ml = milliliter; g = gram; % = percentage; * = there was significant difference between these values (P < 0.05)

### Acute toxicity test

The experimental mice who ingested crude leaf extracts of the three solvents did not show any indication of gross physical or behavioral changes such as hair erection, reduction in feeding and motor activities, weight loss, lacrimation, diarrhea, depression or abnormal secretions within the 24 hrs monitoring period. No fatalities occurred within the observation period of two weeks.

### Anti-malarial activity

#### Effect of crude leaf extracts on PCV and BW

The aqueous, chloroform and methanol leaf extracts of *O. quadripartita* demonstrated a dose-dependent effect on the mean PCV value of *P. berghei-*infected mice (Table 2). With increasing dose percentage change in PCV reduction was lowered for all the three extracts. Also, irrespective of extract type and dose there was reduction on mean PCV on D4 though the difference was significant only for the 200 mg/kg the aqueous, and 200 and 400 mg/kg methanol extracts. But, when the results were compared with the NC only 600 mg of the aqueous extract showed a significant mean PCV reduction (*p* = 0.01).

**Table 2:** Effect of crude aqueous, methanol and chloroform leaf extracts of *O. quadripartita* on PCV and BW of *O. quadripartita* on P. berghei-infected mice

Description	Dose (mg/kg/day)	PCV				BW (g)			
		D0	D4	% change	*p*-value**	D0	D4	% change	*p*-value**
Aqueous	200	60.4 ± 0.60	56.0 ± 0.89^a^	−7.28	0.02, 0.05	31.2 ± 0.37	26.5 ± 0.76 ^a^	−15.06	0.000, 1.000
	400	56.6 ± 1.28	54.0 ± 1.14	−4.59	0.17, 0.34	25.2 ± 0.37	21.8 ± 0.58 ^a^	−13.49	0.000, 0.005
	600	58.6 ± 2.02	56.8 ± 1.98*	−3.07	0.34, 0.01	28.6 ± 0.40	25.1 ± 0.47 ^a^	−12.23	0.000, 0.000
Methanol	200	58.4 ± 1.77	54.2 ± 1.46 ^a^	−7.19	0.02, 0.29	30.6 ± 0.40	26.4 ± 0.24 ^a^	−13.72	0.000, 1.000
	400	57.0 ± 1.60	53.2 ± 1.20 ^a^	−6.67	0.04, 0.59	28.2 ± 0.37	24.6 ± 0.40 ^a^	−12.76	0.000, 0.522
	600	59.4 ± 0.40	55.8 ± 0.73	−6.06	0.06, 0.06	25.6 ± 0.40	23.2 ± 0.37^a^	−09.37	0.000, 0.112
Chloroform	200	57.2 ± 1.59	54.2 ± 1.31	−5.24	0.11, 0.29	27.6 ± 0.24	26.2 ± 0.37*	−05.07	0.027, 0.000
	400	56.4 ± 0.75	55.0 ± 0.54	−2.48	0.46, 0.34	28.6 ± 0.50	30.8 ± 0.58*	+07.69	0.001, 0.000
	600	54.8 ± 1.24	53.8 ± 1.52	−1.82	0.59, 0.17	27.0 ± 0.31	29.6 ± 0.40*	+09.62	0.000, 0.000
dH_2_O (NC)	0.2 ml	56.8 ± 1.93	52.2 ± 1.71 ^a^	−8.09	0.017	29.0 ± 0.31	23.6 ± 0.24 ^a^	−18.62	0.000
T-80 (NC)	0.2 ml	58.0 ± 1.51	52.4 ± 0.92 ^a^	−9.65	0.004	28.8 ± 0.48	23.0 ± 0.44 ^a^	−20.13	0.000
CQ	25	56.8 ± 0.96	56.8 ± 0.89*	0.00	0.674	29.8 ± 0.48	33.2 ± 0.37*^a^	+11.40	0.000

Keys: Values for the packed cell volume (PCV) and body weight (BW) are presented as mean (M) ± standard error of the mean (SEM); n = 5 (number of mice/group in a single experiment); D0: day 0 (day-of-extract-inoculation); D4 = day 4 (the fifth day pos-extract-inoculation; mg/kg/day: 1mg of extract per one kilogram of mice for one day; NC: negative control; T-80 = Tween-80 (NC for chloroform extract); dH_2_O: distilled water (NC for aqueous and methanol extract); CQ: chloroquine phosphate; *: significant compared with that of NC; ^a^: significant difference between D0 and D4. **the first value is for the comparison between D0 and D4, the second is with the NC

A loss in BW was noticed for all groups of *P. berghei-*infected mice four days post-extract-inoculation except for those that ingested 400 and 600 mg/kg chloroform extract. However, the percentage change in BW loss decreased in a dose-dependent manner for all the three extracts at the three doses. In the three groups of infected mice that ingested the three different doses of the aqueous and methanol extracts a significant BW loss was recorded on D4 compared D0, but for the chloroform the difference was not significant. On the other hand; while compared to the NC the change in BW (for all the three doses) was significant for chloroform extract, it was not so for the other two. The infected mice that were treated with 400 and 600 mg/kg chloroform extract significantly gained BW.

#### Effect of crude leaf extracts on parasitaemia

Although there was clear regression of parasitaemia among aqueous or methanol extract-treated mice, compared to the NC, the difference was statistically significant (p < 0.05) only for the methanol extract at 600 mg/kg dose. With regard to the chloroform extract, however, there was significant decline in % parasitaemia at all the three doses in a dose-dependent manner. The maximum parasitaemia reduction was 38.26 % (for the 600 mg) compared to the figure for the NC group (65.4 %). The evidence showed that the chloroform extract, was significantly associated with lowered parasitaemia compared to the NC as well as the other two concurrently tested extracts although the mice had still microscopically detectable parasitaemia.

Correspondingly, in all groups of extract-treated-mice parasitaemia were increasingly chemosuppressed in a dose-dependent manner with the highest suppression being for the chloroform extract - the highest (41.26 %, at 600 mg/kg dose), mild (37.5 %, at 400 mg/kg), and the least (31.7 %, at 200 mg/kg).

Similarly, for the aqueous and methanol extracts all the three doses, except that of aqueous at 200 mg/kg, were correlated with significantly increased mice survival compared to the NC (dH_2_0). The MST of chloroform extract-treated mice was also significantly higher compared to the NC (3 % T-80) groups and it was prolonged in a dose-dependent manner (Table 3).

**Table 3:** Chemosuppressive effect of crude aqueous, chloroform and methanol leaf extracts of *O. quadripartita in P. berghei*-infected mice

Description	Dose (mg/kg/day)	% parasitaemia	*p*-value	% suppression	MST ± SEM	*p*-value
Aqueous	200	63.02 ± 3.91	0.740	0.37	6.2 ± 0.37	0.418
	400	54.62 ± 2.20	0.142	16.53	7.4 ± 0.51*	0.018
	600	51.26 ± 2.05	0.056	21.67	8.2 ± 0.37*	0.001
Methanol	200	55.92 ± 0.84	0.196	14.5	7.4 ± 0.51*	0.018
	400	50.94 ± 1.24	0.510	22.16	8.2 ± 0.37*	0.001
	600	49.48 ± 2.06*	0.033	24.4	8.4 ± 0.24*	0.000
Chloroform	200	44.48 ± 10.18*	0.006	31.7	8.0 ± 1.14*	0.004
	400	40.7 ± 9.66*	0.001	37.5	9.8 ± 0.58*	0.000
	600	38.26 ± 8.78*	0.001	41.26	11 ± 0.63*	0.000
DH_2_O (NC)	0.2 ml	65.44 ± 1.7	-	0.00	5.6 ± 0.24	
T-80 (NC)	0.2 ml	65.14 ± 1.7	-	0.00	5.8 ± 0.37	
CQ	25	0.00	0.000	100	ND	

Keys: mg/kg/day: 1 mg of extract per one kilogram of mice for one day; MST: mean survival time; SEM: standard error of the mean; NC: negative control; T-80 = Tween-80 (NC for chloroform extract); dH_2_O: distilled water (NC for aqueous and methanol extract); CQ: chloroquine phosphate; ND: no death *: significant compared with that of NC

## Discussion

While methanol yielded significantly higher extraction efficiency, the chloroform extract was the lowest. The probable reason for this variation could be due to high concentration of polar compounds in the leaf of the plant species that better dissolve in methanol which is a polar solvent. The quality and quantity of phytochemicals extracted from plant materials differ depending on, among other factors, the solvent type used. The comparative ability of extraction solvents of penetrating the cellular membrane to extract the intracellular ingredients from the plant material may impact extract yield. Some reports show that methanol extracts more number and types of compounds in plant materials than other extraction solvents such as aceton, chloroform, ether, water and ethanol [41]. While chloroform is preferred to extract terpenoids and flavonoids water is employed to solubilize a wide range of plant metabolites like anthocyanins, starches, tannins, saponins, terpenoids, polypeptides and lectins. Methanol releases more diverse phytochemicals such as anthocyanins, tannins, saponins, terpenoids, xanthoxylines, totarol, quassinoids, lectones, flavones, phenols and polyphenols [42].

The finding that the 3 % T-80, which was used as a NC (for the chloroform extract) in the present study and in which the extract was dissolved to deliver to the mice, neither suppressed malaria parasitaemia in the infected mice nor prevented weight loss and PCV depletion indicated lack of possible T-related effect on the malaria parasites.

Oral administration of the methanol, aqueous and chloroform extracts of *O. quadripartita* did not show changes in the general appearance or behavioral pattern of the experimental mice until the end of 14 days. Further, no death was observed in the animals receiving the extract up to a dose of 2000 mg/kg BW, which is about 10 times the minimum effective dose tested (200 mg/kg). If a test substance has a lethal dose (LD_50_) higher than 3 times the minimum effective dose, it can be a good candidate for further studies [43]. Therefore, absence of mortality up to an oral dose of 2000 mg/kg could indicate that the test extracts were safe and this could explain the routine use of the plant by the local people for traditional management of malaria.

Significant BW increase among *P. berghei*-infected mice four days after ingesting 400 and 600 mg chloroform crude leaf extract of *O. quadripartita* compared to the untreated controls suggests the effect of the extract in preventing malaria-related weight loss. It is well-established that BW loss is one feature of murine malaria [44]. The present result is in agreement with other similar studies that reported mice BW loss using different plant products and extraction solvents [45–47]. Lack of significant reduction in the mean BW among aqueous and methanol extract-fed infected mice four days post-treatment further signifies the positive effect of the plant material on mice BW.

The absence of significant PCV reduction among extract-treated mice at the doses of 200 and 400 mg/kg of the aqueous extract may indicate the protective activity of the crude extract. Furthermore, observing a significantly lower PCV reduction among the same groups of mice at the highest dose (600 mg/kg) shows the presence of anti-malarial chemicals in the dose administered. But it appears that the activity of the methanol extract was not strong enough to significantly prevent PCV reduction among *P. berghei-*infected mice. On the contrary, the PCV of chloroform extract-treated mice remained not significantly changed on D4, irrespective of dose, underlining the protective role of the extract against malaria.

The influence of malaria on hematological parameters is extensively investigated and PCV reduction is considered a hallmark of both human and rodent malaria (reviewed in Lamikanra *et al.* [48]. Infected mice may suffer from severe malarial anemia because of rapid erythrocyte destruction, either by parasitaemia and/or spleen reticulo-endothelial cells. For instance, in one study it was noted that within an estimated 48 hrs of post-infection rodent PCV was depleted to 43-44 % [49]. Further, *P. berghei* increased erythrocyte fragility and led to subsequent reduction of PCV in infected-mice [50].

Multiple other *in vivo* studies on rodent malaria using diverse plant species from Ethiopia as well as abroad have reported similar results [6, 45–47, 51, 52]. Scarcity of previous reports pertaining to anti-malarial activity of *O. quadripartita* including the relative composition and predominance of its leaf chemicals could not permit a discussion from comparative perspective. Although the antimicrobial [19, 20] and antiparasitic activities [21] of *O. quadripartita* were investigated to some extent, its plasmodial effect was little explored.

The 4-day suppressive test is a standard test commonly used for *in vivo* anti-malarial phytochemical screening in which ≥30 % reduction in parasitaemia following treatment makes a product to be considered active [36, 43]. Accordingly, the chloroform extract of *O. quadripartita* which showed 31.7 % suppression at the lowest, 37.5 % at the medium and 41.26 % at the highest doses can be classified as active. The dose-dependent variation in chemosuppression could be attributed to the low concentration of schizocidal compounds in natural products and as such their activity may be undetectable in lower doses. This increased percent suppression of parasitaemia with increased dose was observed by other studies on different plant species [45, 47, 53].

However, both the aqueous and methanol extract of *O. quadripartita* didn’t display comparable suppressive activity on *P. berghei* even at the highest dose delivered (600 mg/kg). This at least undetectable level of anti-malarial activity of aqueous and methanol crude leaf extract of *O. quadripartita* may be an indication that the active ingredients extracted by these solvents might have less potent anti-malarial property. This can be explained by the fact that some plants may contain chemicals that are more soluble in polar solvents such as water, ethanol and methanol while others contain chemicals that are more soluble in non-polar solvents such as chloroform. Thus, the crude aqueous or methanol extracts in the current study were higher in terms of yield but they contained little compounds that were efficacious at least against *P. berghei.*

A prolonged MST with significant difference, compared to the NC, was observed for mice treated with all the three extracts regardless of dose except for 200 mg/kg of the aqueous extract implying the role of the plant material in the control of murine malaria. Particularly, the chloroform extract was highly associated with prolonged MST even at the minimum dose implicating the dominant presence of antimalarial bioactive compounds of the plant tissue in this extract.

Nevertheless, the chloroform extract itself was less effective compared to CQ, the standard drug against *P. berghei*. CQ treatment (25 mg/kg) during the infection seemed radically cleared parasitaemia or at least there was no microscopically detectable parasitaemia. Rodent malaria clinical manifestation like diarrhea, lethargy, piloerection, reduced locomotor activity, *etc.* were non-existent among the CQ-treated showing that the parasitological cure was clinical as well. Mice were appearing and acting healthy on day 28. The undetectable level clearance of parasitaemia following the CQ chemotherapy indicates that the *P. berghei* strain used in the study was highly sensitive to the drug and lends support that this rodent malaria model system remains effective for *in vivo* anti-malarial testing.

## Conclusions

When orally administered, no adverse effects were noted for the plant extracts ranging from 1000-2000 mg/kg doses signifying the safety of the extracted phytochemicals in mice via the oral route. The aqueous and methanolic extracts showed some parasite suppressive effects on *P. berghei-*infected mice in a dose-related fashion and the chloroform extract was observed to have the strongest activity although its effect was not comparable to that of the standard drug which achieved parasite clearance to undetectable level microscopically. The anti-plasmodial activity as well as lack of toxicity of the crude extract found in the present study may partly confirm the claim by traditional practitioners about the use of the plant against malaria. However, the finding is only preliminary; and thus confirmatory studies followed by isolation and characterization of the active anti-malarial compound (s) of the plant that is/are responsible for the observed parasite suppression thereby resulting in increased MST, BW loss prevention and PCV reduction in the *P. berghei*-infected mice are recommended.

## Abbreviations

AAU: Addis Ababa University
ANOVA: Analysis of Variance
BW: Body Weight
CQ: Chloroquine
D0: Day zero
D4: Day four
dH_2_O: Distilled water
DMCMB: Department of Microbial, Cellular and Molecular Biology
LD: Lethal Dose
M ± SEM: Mean plus or minus Standard Error of the Mean
MST: Mean Survival Time
NC: Negative Control
ND: No Death
OCED: Organization for Economic Cooperation and Development
PCV: Packed Cell Volume
% parasitaemia: Percentage Parasitaemia
% suppression: Percentage Suppression
rpm: revolutions per minute
T-80: Tween-80
WHO: World Health Organization
